# Switchable THz Bi-Functional Device for Absorption and Dual-Band Linear-to-Circular Polarization Conversion Based on Vanadium Dioxide–Graphene

**DOI:** 10.3390/s25123644

**Published:** 2025-06-10

**Authors:** Yiqu Wang, Haohan Xie, Rong Liu, Jun Dong

**Affiliations:** 1 School of Physics and Electronics, Hunan University, Changsha 410082, China; yiquwang898@outlook.com (Y.W.); rongliu@hnu.edu.cn (R.L.); 2 College of Information Science and Engineering, Hunan Normal University, Changsha 410081, China

**Keywords:** bi-functional THz device, graphene, dioxide absorption, linear-to-circular polarization conversion

## Abstract

This academic paper proposes a terahertz (THz) device featuring dynamic adjustability. This device relies on composite metamaterials made of graphene and vanadium dioxide (*VO*_2_). By integrating the electrically adjustable traits of graphene with the phase transition attributes of *VO*_2_, the suggested metamaterial device can achieve both broadband absorption and dual-band linear-to-circular polarization conversion (LCPC) in the terahertz frequency range. When *VO*_2_ is in its metallic state and the Fermi level of graphene is set to zero electron volts (eV), the device shows remarkable broadband absorption. Specifically, it attains an absorption rate exceeding 90% within the frequency span of 2.28–3.73 terahertz (THz). Moreover, the device displays notable polarization insensitivity and high resistance to changes in the incident angle. Conversely, when *VO*_2_ shifts to its insulating state and the Fermi level of graphene stays at 0 eV, the device operates as a highly effective polarization converter. It attains the best dual-band linear-to-circular polarization conversion within the frequency ranges of 4.31–5.82 THz and 6.77–7.93 THz. Following the alteration of the Fermi level of graphene, the device demonstrated outstanding adjustability. The designed multi-functional device features a simple structure and holds significant application potential in terahertz technologies, including cloaking technology, reflectors, and spatial modulators.

## 1. Introduction

Metamaterials are a class of artificially designed composite materials that consist of subwavelength structural units. Generally, the sizes of these elements are less than the wavelength of the waves they engage with. As a result, the physical characteristics of metamaterials are mainly dictated by their structure instead of the material makeup of the constituent materials. These attributes, like total absorption [1], irregular reflection [2], and negative refractive index [3], are not present in natural materials. Metamaterials have been extensively utilized across diverse domains, such as stealth technology [4] and perfect lenses [5,6]. The functionality of terahertz (THz) metamaterials is predominantly determined by their configuration, especially the arrangement of the unit cell. Once fabricated, the optical properties of early optical metamaterials are difficult to modify. However, with the advancement of metamaterial technology and their increasing integration with tunable materials, the development of switchable multi-functional optical devices has become possible [7].

Recently, tunable metamaterials incorporating materials such as doped silicon [8], germanium antimony telluride (GST) [9,10,11,12], graphene [13,14,15,16,17], and vanadium dioxide (*VO*_2_) [18,19,20,21] have been introduced to achieve reconfigurability. Graphene, a planar, two-dimensional substance made up of carbon atoms featuring sp^2^ hybridized orbitals, demonstrates distinctive electrical and optical characteristics. These characteristics include transparency, the quantum Hall phenomenon, high electrical conductivity, and an adjustable band structure. This tunable band structure can be adjusted through chemical doping or electric field effects, allowing for precise control over graphene’s electrical characteristics, including the bandgap and carrier concentration. Consequently, metamaterials crafted from graphene serve as an optimal option for electromagnetic devices that can be adjusted and switched. Consider, for example, a programmable graphene metasurface. This metasurface, which functions based on a principle similar to electromagnetically induced transparency, is created by altering the bias voltage applied to the graphene patterns linked to dark resonators [15]. By controlling the Fermi level of graphene, both wide-band linear-to-linear polarization conversion (LPC) and linear-to-circular polarization conversion can be efficiently accomplished [17].

At the same time, *VO*_2_ is a material that is distinctive for its remarkable phase transition characteristics [22]. As a metal oxide, it undergoes a reversible transformation from a metal to an insulator at around 68 °C. This transition results in substantial alterations in key properties such as electrical conductivity, light absorption, dielectric constant, and lattice structure, offering great potential for diverse technological applications. This paper presents an innovative dual-function design that seamlessly integrates a broadband absorber and a broadband dual-band polarization converter. The proposed structure leverages a reconfigurable metasurface, which exploits the insulator-to-metal phase transition of vanadium dioxide to achieve tunable electromagnetic responses [20,23]. Therefore, integrating the properties of *VO*_2_ and graphene in a single design offers an effective approach to achieve multi-functional, switchable, and reconfigurable absorbers [24]. In an exceptional research endeavor, scientists developed a tunable dual-band terahertz (THz) absorber by combining *VO*_2_ and graphene. By taking advantage of the adjustable characteristics of graphene and the phase transition traits of *VO*_2_, this device facilitated the transition between low-frequency and high-frequency broadband absorption [25]. Further advancements led to the creation of active, switchable, multi-functional metamaterials. For instance, a system combining *VO*_2_ and graphene demonstrated remarkable performance; upon *VO*_2_’s shift to its metallic state, the metamaterial functioned as a multi-band ideal absorber. It demonstrated polarization independence and high resilience to variations in incident angles [26]. Behnaz Bakhtiari and her colleagues put forward a dual-function broadband adjustable terahertz polarization converter metasurface. The constructed structure has a combined setup of electromagnetic materials, allowing for adjustable polarization conversion in both the reflection and transmission modes [27]. These works underscore a broader trend in metamaterials research: the pursuit of tunable, multi-functional designs that can switch between different states and broaden their operational bandwidths [28,29,30,31,32]. While significant progress has been made, achieving a wider operational range during function switching remains a substantial challenge.

Within this conceptual model, we put forward a dual-function apparatus that functions within the terahertz frequency domain. This apparatus is not only adjustable, but also able to switch its functions. By integrating the phase transition attributes of *VO*_2_ with the electro-optical adjustability of graphene, this design surmounts the restrictions of traditional single-function materials. The apparatus is composed of an upper stratum of *VO*_2_, a polyimide thin sheet, and a *VO*_2_ foundation. When vanadium dioxide is in its metallic phase, the device attains an absorption efficacy surpassing 90% within the frequency range of 2.28–3.73 terahertz. The reflectivity of the absorber can be finely tuned by adjusting the Fermi level of graphene. When *VO*_2_ enters its insulating phase, the device facilitates dual-band linear-to-circular polarization conversion in the 4.31–5.82 THz and 6.77–7.93 THz frequency ranges. This device holds significant promise for advanced applications in terahertz multi-functional nanophotonics.

## 2. Theory and Method

**Figure 1:**
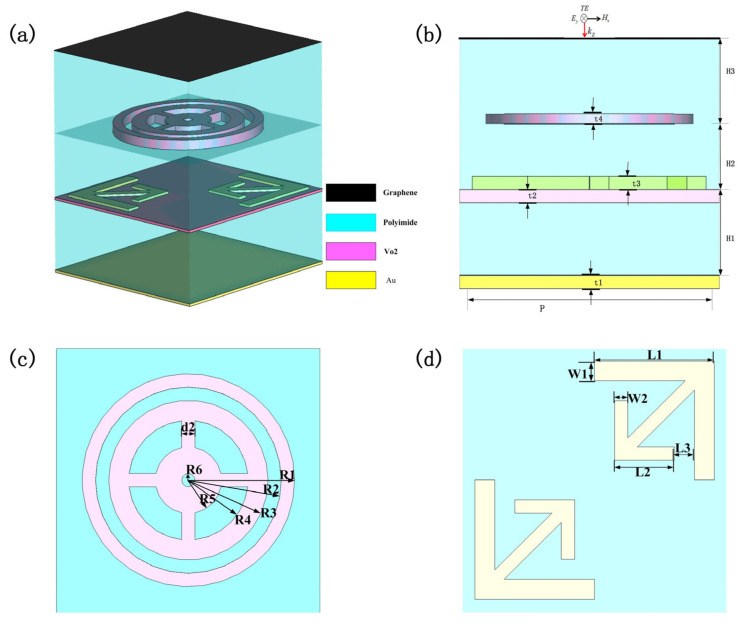
(**a**) Schematic of designed metamaterial; (**b**) side view of the periodic unit structure; (**c**) top view of the second floor; (**d**) top view of the third floor.

Figure 1 presents a schematic diagram of the proposed switchable metamaterial. Beginning from the top-most layer and progressing towards the bottom-most layer, it is composed of a single graphene sheet, a polyimide (PI) spacer, a *VO*_2_ surface pattern, metallic gold segments, a thin *VO*_2_ coating, another polyimide (PI) spacer, and a metallic substrate. This metamaterial is manufactured on a gold base and is engineered to efficiently impede the passage of incoming waves. Significantly, the thickness of the bottom metallic layer is greater than the skin depth, enabling it to act as an optimal reflector. As a result, the supposition of a perfect conductor can be made.

The following content concerns the definitions of the geometric parameters. Along both the x- and y-axes, the periodicity, denoted by *P*, measures 20 µm. The thickness of the metal base, represented as *t*_1_, is 0.2 µm. Likewise, the thickness of the *VO*_2_ thin layer, labeled as *t*_2_, is also 0.2 µm. We will now shift our focus to the measurement of the *VO*_2_ surface configurations. The specified values are as follows: the dimension labeled *R*_1_ measures 8 µm, *R*_2_ has a measurement of 7 µm, *R*_3_ is 6 µm in size, *R*_4_ amounts to 4.5 µm, *R*_5_ is 2.5 µm, and *R*_6_ has a value of 0.5 µm. The spacing, denoted as *d*_2_, is 1 µm. The vertical extent, represented by *t*_4_, attains a value of 0.75 µm. Concerning the metallic elements, the lengths of these elements are *L*_1_ = 9 µm and *L*_2_ = 4.5 µm, respectively. The widths corresponding to these lengths are *W_1_* = 1.5 µm and *W* = 1 µm. Moreover, the thickness of the metallic base, which is denoted as *t*_3_ on this occasion, measures 0.2 µm. Ultimately, the thicknesses of the polyimide (PI) spacer layers are as follows: *H*_1_ measures 5.5 µm, *H*_2_ is 5 µm thick, and *H*_3_ has a thickness of 6.5 µm.

In the case where the incident electromagnetic wave operates in the transverse electric (TE) mode, Figure 1b depicts the orientations of the electric field (E-field) and magnetic field (H-field) within the wave. In this mode, the E and H fields are oriented along the y- and x-axes, respectively. Owing to its structural symmetry, this design is also applicable to the transverse magnetic (TM) mode. Under this specific circumstance, the directions of the electric and magnetic fields are, respectively, reversed along the x-axis and y-axis. The relative permittivity of *VO*_2_ can be accurately characterized by applying the Drude model [33],

(1) $$\varepsilon_{{VO}_{2}}\left(\omega \right)=\varepsilon_{\infty }-\frac{\omega_{P}^{2}\sigma_{VO_{2}}}{\omega^{2}+i\gamma \omega }$$

where $\varepsilon_{\infty }=12$ denotes the permittivity at very high frequencies. The plasma frequency is expressed as follows:

(2) $$\omega_{p}^{2}\left(\sigma_{{VO}_{2}}\right)=\left(\frac{\sigma_{{VO}_{2}}}{\sigma_{0}}\right){\cdot \omega }_{p}^{2}\left(\sigma_{0}\right)$$

The value of the square of the plasma frequency, denoted as $\omega_{p}^{2}(\sigma_{0})$, is $1.4\times 10^{15}$ radians per second. Moreover, the damping rate, symbolized by γ, equals $5.75\times 10^{13}$ radians per second, and the conductivity $\sigma_{0}$ is $3\times 10^{5}$ siemens per meter. *VO*_2_ is a thermally sensitive substance. It undergoes a transition from an insulating phase to a metallic phase at a critical temperature of around 340 Kelvins.

**Figure 2:**
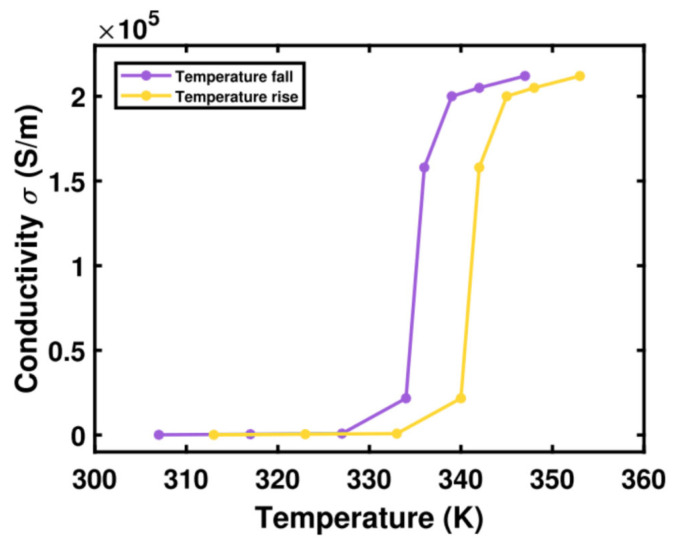
The conductivity of *VO*_2_ as a function of temperature extracted from data [34].

The conductivity changes of *VO*_2_ in relation to temperature during the heating and cooling processes were sourced from references [34]. These data are then presented in Figure 2. Evidently, temperature fluctuations have a substantial impact on the conductivity of *VO*_2_, particularly at the phase transition point. Owing to the thermal hysteresis phenomenon, there are minor disparities between the transition curves of the heating and cooling cycles. Nevertheless, this does not influence the shift between the two stable phases. Using the graph in Figure 2 and Equation (1), one can calculate the relative permittivity of *VO*_2_ at various temperatures.

The surface conductivity of graphene is described by the Kubo equation [35]:

(3) $$\begin{array}{c} \sigma_{g}\left(\omega \right)=\sigma_{intter}+\sigma_{intra}\\ \;\;\;\;\;\;=\frac{2e^{2}k_{B}T}{\pi \hbar }\cdot \frac{j}{-\omega +j\tau^{-1}}\cdot \ln\left[2\cdot \cosh\left(\frac{E_{f}}{2k_{B}T}\right)\right]\\ \;\;\;\;\;\;-\frac{je^{2}}{4\pi \hbar }\cdot \ln\left[\frac{2E_{f}-\hbar \left(\omega -j\tau^{-1}\right)}{2E_{f}+\hbar \left(\omega -j\tau^{-1}\right)}\right] \end{array}$$

One might wonder about the location of the electron charge. Here, $E_{f}$ stands for the Fermi energy level, ℏ stands for the reduced Planck constant, and $k_{B}$ signifies the Boltzmann constant. ω, equivalent to 2πf, denotes the angular frequency. The temperature is set at *T* = 300 K, and τ represents the relaxation time. The surface conductivity of graphene consists of intra-band conductivity (the first term in Equation (3)) and inter-band conductivity (the second term in Equation (3)). At low frequencies, the inter-band term dominates, and when $E_{f}\gg k_{B}T$, the conductivity $\sigma_{g}$ can be approximated by the Drude model as

(4) $$\sigma_{g}\left(\omega \right)=\frac{e^{2}}{\pi \hbar }\cdot E_{f}\cdot \tau \cdot \frac{1}{1+i\omega \tau }$$

In the realm of computational modeling, graphene is represented as a planar structure with a thickness denoted as $h_{g}$, which is equal to 0.345 nanometers. The dielectric constant of this material, represented as $\epsilon_{g}$, is defined by the formula $\epsilon_{g}=\epsilon_{0}-j\frac{\sigma_{g}(\omega )}{h_{g}(\omega )}$. Significantly, the electrical conductivity of graphene can be adjusted in a dynamic manner by modifying the Fermi level.

## 3. Results and Discussion

### 3.1. Broadband Absorber

In the metallic phase of *VO*_2_, the absorber is composed of an upper structure made of vanadium dioxide, a polyimide layer, and a base of vanadium dioxide. The absorption rate, denoted as A(ω), can be calculated using the equation A(ω)=1−R(ω)−T(ω). In this equation, $R(\omega )={\left|S_{11}\right|}^{2}$ represents the reflectance, while $T(\omega )={\left|S_{21}\right|}^{2}$ stands for the transmittance. Within the simulated frequency realm, the lower metallic layer possesses an adequate thickness that exceeds the material’s skin depth. As a result, the transmissivity is nearly zero. Consequently, the absorption rate can be simplified to $A(\omega )={1-|S_{11}|}^{2}$.

**Figure 3:**
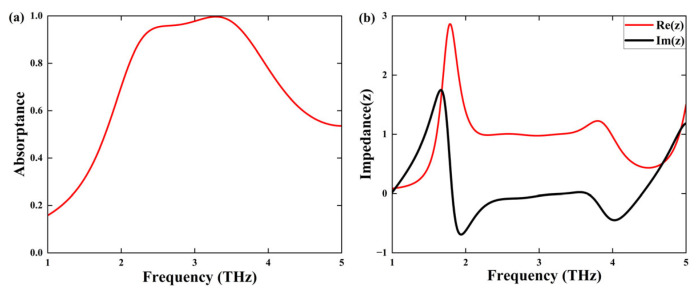
(**a**) Absorptance of absorber when *VO*_2_ is in the metal phase; (**b**) relative impedance of the absorber.

The absorption spectrum obtained from this work is presented in Figure 3a. The broadband absorption spectrum vividly shows that the absorber attains outstanding absorption efficiency for electromagnetic waves within the frequency span of 2.28–3.73 THz. It has a bandwidth of roughly 1.45 THz, and the absorption ratios exceed 90%. Significantly, within the frequency range from 3.00 to 3.47 THz, the absorber reaches an impressive 98% absorption rate. Moreover, this research paper presents the notion of relative impedance to evaluate the outcomes. The formula for relative impedance is as follows [36,37]:

(5) $$Z=\pm \sqrt{\frac{1+S_{11}^{2}-S_{21}^{2}}{1-S_{11}^{2}-S_{21}^{2}}}$$

As depicted in Figure 3b, within the frequency interval spanning from 2.28 to 3.73 THz, the imaginary component of the absorber’s relative impedance hovers near zero. Concurrently, the real component steadily remains at around one. Under these circumstances, the characteristic impedance of the absorber is roughly equivalent to the impedance of free space, thereby attaining impedance matching. This impedance matching phenomenon substantially diminishes the reflection of the incident wave, resulting in a greater absorption rate.

**Figure 4:**
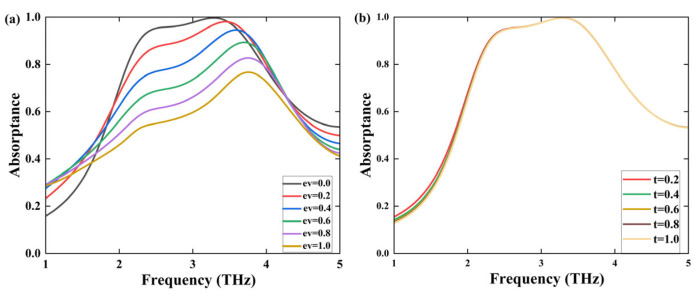
(**a**) Effects of different chemical formulas of graphene on absorption rate; (**b**) the relationship between graphene’s hesitation time t and absorption rate.

As shown in Figure 4a, it can be clearly seen that the absorber consistently attains an energy absorption ratio surpassing 90% in the frequency interval from 2.28 to 3.73 THz. However, a variation in the chemical composition of graphene correlates with a gradual decline in absorption efficiency. This alteration can significantly impact the electronic structure of graphene, specifically affecting key parameters such as carrier concentration and mobility. A reduced carrier concentration can lead to the enhanced reflection of electromagnetic waves, diminishing their absorption. Furthermore, alterations to the chemical makeup can also have an impact on the surface unevenness of graphene. This surface unevenness is of great importance as it is closely linked to the reflection and dispersion of electromagnetic waves. Increased surface roughness can result in greater scattering, thus enhancing reflection. This interplay between chemical composition and surface characteristics is crucial for optimizing the absorber’s performance [38].

**Figure 5:**
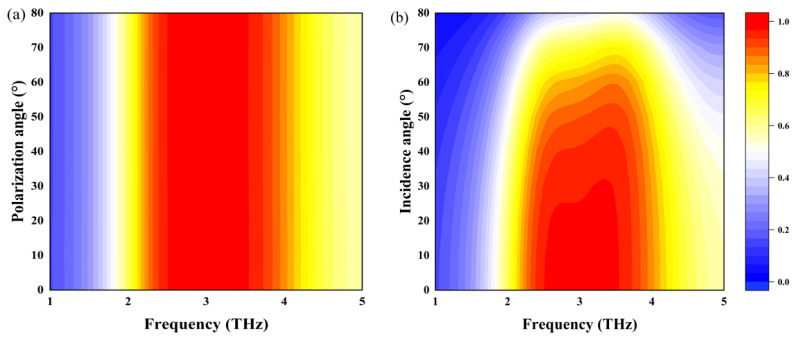
(**a**) Relationship between polarization angle and absorptivity at normal incidence; (**b**) relationship between incident angle and absorptivity.

In the preceding discussion, we examined the characteristics of the absorber under normal incidence. However, it would be advantageous to design an absorber that can operate effectively under oblique incidence, thus emphasizing the significance of investigating its angular performance. Figure 5a shows that when the polarization angle ranges from 0° to 80°, there is a change in the absorption rate. This implies that polarization has a minimal influence on the overall functionality of the absorber. This polarization insensitivity arises from the intrinsic symmetry of the absorber. As the incident angle increases, the operating bandwidth and intensity of the incident wave remain remarkably stable up to 50°, where absorption efficiency still reaches 90%, as depicted in Figure 5b. However, when the incident angle exceeds 50°, the performance begins to deteriorate due to the reduction in the strength of the magnetic field’s parallel component.

This angular insensitivity is a result of the strong coupling between localized surface plasmon resonances and the incident wave. Beyond a critical incident angle, this coupling weakens dramatically, leading to a substantial decrease in absorption rate at 60° incidence, as shown in Figure 5b. In summary, the absorption rate of the absorber remains polarization-independent under normal incidence. Furthermore, it maintains a high absorption rate and exhibits polarization insensitivity within the incident angle range of 0° to 50°. This combination of attributes underscores the absorber’s potential for practical applications in diverse conditions.

### 3.2. Linear-to-Circular Polarization Converter

During the insulating phase of *VO*_2_, the linear-to-circular polarization converter is composed of two metallic patches, a polyimide layer, and a gold substrate. The simulations of this converter were carried out utilizing the Finite Integration Technique (FIT), with the application of floating ports and periodic boundary conditions. An incident flux port represents a wave with transverse electric (TE) polarization that propagates in the negative z-axis direction. The reflection coefficients for the reflected waves polarized in the x-axis and y-axis directions are labeled as Rxy and Ryy, respectively. Owing to the symmetry of the top-layer structure along the diagonal, when incident waves with x-polarization and y-polarization interact with the polarization converter, the co-polarized and cross-polarized reflection coefficients stay identical. When a y-polarized wave strikes the quasi-spherical surface perpendicularly, the electric fields of both the incident wave and the reflected wave can be represented as follows:

(6) $$\vec{E_{i}}=\left\{\begin{array}{c} E_{xi}\vec{x},\;for\;the\;\mathrm{x-component}\\ E_{yi}\vec{y},\;for\;the\;\mathrm{y-component} \end{array}\right.$$

(7) $$\vec{E_{r}}=\left\{\begin{array}{c} E_{xr}\vec{x},\;for\;the\;\mathrm{x-component}\\ E_{yr}\vec{y},\;for\;the\;\mathrm{y-component} \end{array}\right.$$

Within this context, $E_{xi}(E_{xr})$ and $E_{yi}(E_{yr})$ denote, respectively, the electric field constituents of the incident (reflected) wave along the x-axis and y-axis. The formulation for the reflected wave is as follows:

(8) $$\vec{E_{r}}=\sum_{i=x,y}R_{yi}\cdot e^{-j\left(kz-\phi_{yi}\right)}\cdot E_{yi}\cdot {\vec{y}}_{i}$$

Within this context, *R_xy_* and *R_yy_* represent the co-polarization and cross-polarization reflection coefficients, respectively. To assess the polarization conversion capacity of the proposed converter more effectively, the Polarization Conversion Ratio (PCR) is presented to characterize the efficiency of polarization conversion. Its definition is as follows:

(9) $$PCR\left(x\right)=\frac{{\left|r_{yx}\right|}^{2}}{{\left|r_{xx}\right|}^{2}+{\left|r_{yx}\right|}^{2}}$$

(10) $$PCR\left(y\right)=\frac{{\left|r_{xy}\right|}^{2}}{{\left|r_{xy}\right|}^{2}+{\left|r_{yy}\right|}^{2}}$$

The phase’s difference is defined as

(11) $$\phi_{diff}=\phi_{xy}{-\phi }_{yy}$$

**Figure 6:**
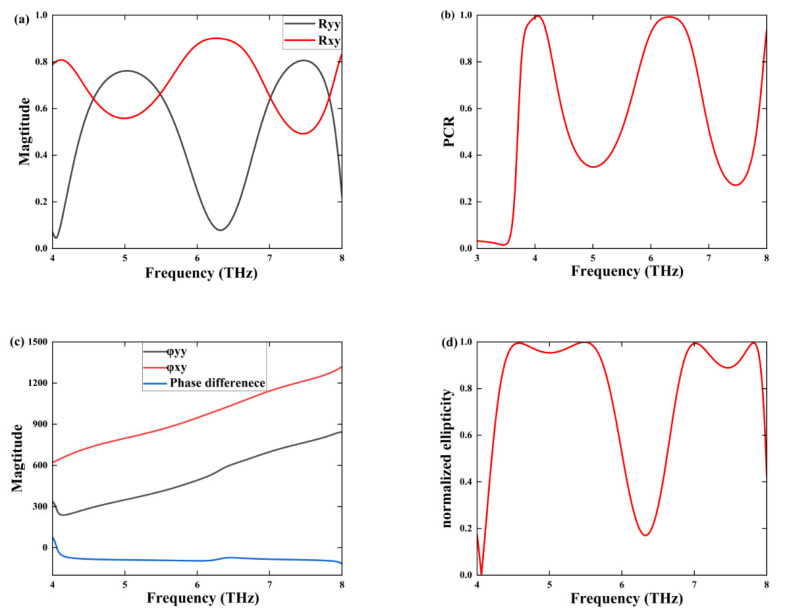
(**a**) Reflection coefficients of the metamaterial; (**b**) PCR; (**c**) *ϕ_xy_* *ϕ_yy_* and phase difference; (**d**) the normalized ellipticity.

Figure 6c reveals that the magnitudes of the reflection coefficients $\left|R_{xy}\right|$ and $\left|R_{yy}\right|$ are nearly equivalent, with a phase difference ϕ-diff of approximately −90°, occurring within the frequency ranges of 4.31–5.82 THz and 6.77–7.93 THz. Furthermore, the normalized ellipticity *χ*, which serves as a quantitative measure of the linear-to-circular polarization conversion efficiency, is observed to be nearly 90% within the same frequency bands, as illustrated in Figure 6d. This significant value of χ demonstrates the exceptional ultrawideband performance of the LTC polarization converter. The normalized ellipticity χ is defined by the following formula: $\left(A=\left|R_{yy}\right|,\;B=\left|R_{xy}\right|,\;C=phi-diff\;\right).$

(12) $$\chi =2\cdot \frac{AB}{A^{2}{+B}^{2}}\cdot \sin\left(c\right)$$

To demonstrate the change in polarization of the reflected wave, the Stokes parameters are as follows [39]:

(13) $${I=A}^{2}{+B}^{2}$$

(14) $$Q=A^{2}{-B}^{2}$$

(15) $$U=2\cdot AB\cdot \cos\left(c\right)$$

(16) $$V=2\cdot AB\cdot \sin\left(c\right)$$

To further analyze the circular polarization reflection field, we introduce the polarization azimuth angle *α* and the ellipticity angle *β*, as expressed in the following equation [40]:

(17) α=12·tan−1⁡2ABA2−B2·cos⁡c

(18) β=12·sin−1⁡2ABA2+B2·sin⁡c

**Figure 7:**
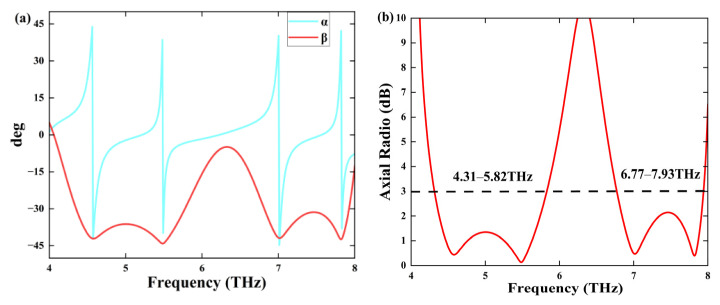
(**a**) The polarization azimuth angle *α*, the ellipticity angle *β*; (**b**) the aspect ratio *AR*.

In this study, we meticulously analyze the orientation of the ellipse through the parameter α, which signifies its deviation from the principal axis, while β effectively captures the ellipse’s divergence from a standard circle. Figure 7a presents β values at frequencies of 4.57 THz, 5.47 THz, 7.01 THz, and 7.81 THz, recorded as −42.2°, −44.2°, 42.1°, and 42.5°, respectively, highlighting a compelling alignment with the standard circle at 45°. As a result, the reflected waves exhibit characteristics that closely approximate circular polarization, albeit not entirely. The angle of reflection can be precisely defined as

(19) $$AR=10\cdot \log_{10}\left(\tan\left(\beta \right)\right)$$

Figure 7b illustrates that within the frequency ranges of 4.31–5.82 THz and 6.77–7.93 THz, AR is less than 3 dB. All polarization ellipses with AR values below 3 dB can be approximated as circular.

**Figure 8:**
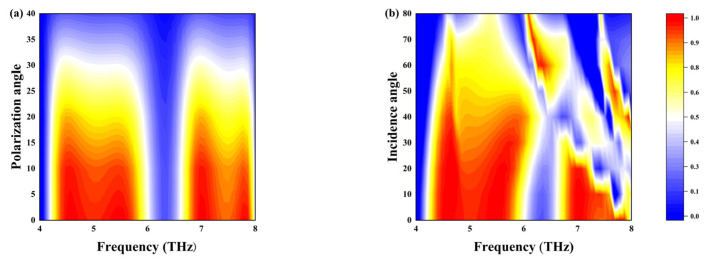
(**a**) The relationship between polarization angle and normalized ellipticity; (**b**) the relationship between the angle of incidence and normalized ellipticity.

Figure 8a illustrates that the polarization angle significantly affects the transition from linear-to-circular polarization. As the polarization angle increases, the normalized ellipticity decreases. In particular, when the polarization angle is 0°, the normalized ellipticity is nearly 0. Figure 8b depicts the effect of the angle of incidence (varying from 0° to 80°) on the normalized ellipticity. It is evident that within the frequency span of 4.31 to 5.82 THz, the device shows virtually no responsiveness to the angle of incidence. However, between 6.77 and 7.93 THz, the angle of incidence substantially affects the normalized ellipticity. This indicates that the angle of incidence has minimal impact on the first frequency band, but a more significant impact on the second frequency band.

Table 1 shows a contrast with results reported in the past to emphasize the benefits of the engineered metamaterials. Unlike traditional absorption structures that use tunable *VO*_2_ in the metallic component, the proposed design incorporates graphene and *VO*_2_, enabling a dual-band operating bandwidth. This configuration allows for efficient switching between absorption and polarization conversion by simply varying the temperature. The findings derivable from the comparison indicate that the synthetically designed metamaterials present a wider range of operational frequencies and greater efficiency. This trait renders them highly appropriate for practical applications in the real world.

## 4. Conclusions

In this research, we have put forward a dual-purpose metamaterial device that relies on *VO*_2_ and graphene. When *VO*_2_ is in its metallic state, the suggested metamaterial device attains a broadband absorption of over 90% within the frequency interval of 2.28–3.73 terahertz. This metamaterial exhibits insensitivity to the incident angle and maintains broadband absorption even at high incident angles. When *VO*_2_ transitions to its insulating phase, the metamaterial functions as a linear-to-circular polarization converter within the frequency bands of 4.31–5.82 THz and 6.77–7.93 THz. The findings imply that adjusting the Fermi level of graphene can regulate the absorption efficiency of the metamaterial. This work offers a novel approach to developing high-performance, broadband, and multi-functional terahertz metamaterials, with promising applications in fields such as cloaking technology, electromagnetics, and radar communication, and paving the way for future practical advancements.

## Figures and Tables

**Table 1:** Comparison of metamaterials’ performances.

Ref.	Tunable Material	Absorption		Polarization Conversion	
		Absorption (THz)	Bandwidth (THz)	PC (THz)	Bandwidth (THz)
[20]	VO_2_	0.52–1.20 (>90%)	0.68	0.42–1.04	0.62
[29]	VO_2_	0.68–1.60 (>90%)	0.92	0.82–1.60	0.78
[17]	Graphene	NO	NO	2.89–3.34	2.89–3.34
				3.34–3.59	3.34–3.59
[30]	Graphene	NO	NO	0.60–0.67	0.07
				0.72–0.97	0.27
[31]	Graphene and VO_2_	1.38–3.16 (>90%)	1.78	3.76–4.01	0.25
[32]	Graphene and VO_2_	NO	NO	1.57–2.74	1.17
				1.13–1.59	0.46
This work	Graphene and VO_2_	2.28–3.73 (>90%)	1.45	4.31–5.82	1.51
				6.77–7.93	1.16

## Data Availability

The data that support the findings of this study are available from the corresponding author upon reasonable request.
